# Biology of the Two-Spotted Spider Mite on Strawberry Plants

**DOI:** 10.1007/s13744-013-0184-7

**Published:** 2014-01-11

**Authors:** L B Monteiro, T M A Kuhn, A F Mogor, E D B da Silva

**Affiliations:** Lab Manejo Integrado de Pragas, Depto de Fitotecnia e Fitossanitarismo, UFPR, 82210-280 Curitiba, PR Brasil

**Keywords:** Development, oviposition, strawberry, susceptibility, *Tetranychus urticae*

## Abstract

The two-spotted spider mite, *Tetranychus urticae* Koch, is commonly found on strawberry crops (*Fragaria* x *ananassa*). Strawberry plants have defensive mechanisms, which in turn influence the behavior of herbivores. The oviposition and development of the two-spotted spider mite were evaluated on the leaf disks of the cultivars ‘Aromas,’ ‘Camarosa,’ ‘Camino Real,’ ‘Diamante,’ ‘Diamante 10,’ ‘Diamante 50,’ ‘Festival,’ and ‘Seascape.’ It was observed that on cultivars such as ‘Aromas,’ ‘Camarosa,’ and ‘Seascape,’ immature survivorship was higher, but no difference was found during the developmental period from egg to adult of *T. urticae*. The immature development time was also longer on ‘Camarosa.’ Females laid more eggs on ‘Seascape’ (8.4 eggs/day), and the least on ‘Camarosa’ (1.0 egg/day). Mortality was higher at the larval stage and reached more than 50% in three cultivars ‘Camarosa,’ ‘Diamante,’ and ‘Seascape.’ Thus, the cultivars ‘Camarosa,’ ‘Diamante,’ and ‘Seascape’ were the ones that mostly affected the survival, development, and reproduction of *T. urticae*.

## Introduction

The breeding process of a given cultivar influences several metabolic and morphological mechanisms of resistance in the search for plants with high productivity and quality fruits. The selection of plants, which are resistant to herbivores, may lead to fitness costs influencing the growth of plants and reproduction (Strauss *et al* 2002).

Plant resistance to herbivores is associated with induced (Kielkiewicz 1988, Thaler & Karban 1997) and constitutive (Lourenção *et al* 2000) factors. Although there are a variety of factors influencing plant resistance to herbivores (Bergelson & Purrington 1996), genetic variables are the basis for the selection of resistant populations (Thaler & Karban 1997). Even though the majority have explored the relationships between constituent and induced resistance (Gianoli 2002, Traw 2002, Fadini *et al* 2004), some have evaluated the effect of plant resistance on the fitness and behavior of herbivores (Thaler & Karban 1997, Underwood & Rausher 2000).

The two-spotted spider mite, *Tetranychus urticae* Koch (Acari: Tetranychidae), is a cosmopolitan, polyphagous species, and it is a pest of strawberry crops. Its control is exclusively based on the use of acaricides, and less susceptible cultivars are required to reduce the impact of the use of pesticides (Lourenção *et al* 2000, Fadini *et al* 2004). Therefore, we evaluated the development and reproduction of two-spotted spider mite on eight strawberry cultivars under laboratory conditions.

## Material and Methods

The strawberry cultivars ‘Aromas,’ ‘Camarosa,’ ‘Camino real,’ ‘Diamante,’ ‘Diamante 10,’ ‘Diamante 50,’ ‘Festival,’ and ‘Seascape’ were evaluated. Seedlings were obtained from the BioAgro Company (Araucária, PR) and established in 5-l containers filled with aerated and fertilized soil, according to Raij *et al* (1996), and placed in a greenhouse.

### *Rearing methods*

A colony of the two-spotted spider mite has been multiplied on bean plants (*Phaseolus vulgaris*) since 1991, according to Monteiro (2002), and kept in a greenhouse. Females with the same age were obtained, placed on leaf disks of beans (20 mm Ø), and the disks were arranged on moistened cotton in an acrylic box (Monteiro 2001). Five females were allowed to oviposit for 24 h, and the development of the newly hatched mites was followed until the adulthood.

### *Assessment of reproduction*

Developing strawberry leaves were collected and transferred to the laboratory in thermal bags at 10°C. Fifty leaf disks (10 mm Ø) were cut from each cultivar and are placed in boxes (13 × 8.5 × 3 cm) with moist cotton as described earlier. We adopted a completely randomized experimental design.

A 24-h-old female was placed on each disk and kept under controlled conditions (25 ± 1°C; 70 ± 10% RH; and 14-h photophase). The number of eggs laid was evaluated daily until the female death; eggs were removed to avoid double counting.

### *Two-spotted spider mite development*

Egg laying of individual 24-h-old mated females were assessed for 6 h on each of the 50 leaf disks of each strawberry cultivar and the control plant (*P. vulgaris*). The laid eggs were counted, and their development was monitored at every 8 h. The evaluated developmental stages for both male (M) and female (F) were as follows: egg phase (ED), larval phase (LD), protochrysalis (PCD), protonymph (PND), deutochrysalis (DCD), deutonymph (DN), teleiochrysalis (TCD), and adult development (AD) {male (M), and female (F)}. The observations were carried out until the death of the adult mites. The leaf disks were replaced every 10 days. The experimental conditions were the same as those used to evaluate the oviposition.

Leaf disks of bean plants were used as control plants, as this host was justified due to the costs of adaptation that can occur when mites produced on a given host begin to feed on another host, with consequences to their growth during the immature phases (Jesiotr 1979). Since the *T. urticae* population had been reared on bean plants for 16 years in our laboratories, we expected that this mite had adapted to this host plant. Consequently, we expected a much higher fitness on this plant.

### *Statistical analysis*

Statistical analyses were carried out using the Statgraphics Centurion XV version 15.1.02 (StatPoint®) and SigmaPlot 10 (Systat SoftWare®) programs. The data were transformed using log (x_0.5) before ANOVA, and means were compared using the Tukey test (α = 0.05) or were evaluated using an exponential nonlinear regression.

## Results and Discussion

### *Two-spotted spider mite development*

The period of ED was significantly higher (*F* = 22.5, *df* = 8, *p* = <0.001) on ‘Camino Real’ than on ‘Diamante 10’ and ‘Diamante 50’ (Table 1). The average ED from all cultivars was 30% greater than that obtained in the control. LD was significantly longer (*F* = 7.43, *df* = 8, *p* = <0.001) in ‘Camarosa’ (Table 1), nearly 2.2 times longer than the LD on the control disks. The cultivars ‘Camino Real,’ ‘Festival,’ ‘Diamante 10,’ and ‘Diamante 50’ presented the shortest LD. The variance in the control confirms that the mites were better adapted to the bean host, and that LD was affected in most cultivars, except for ‘Diamante 50.’

**Table 1:** Immature development time and longevity (h) (±se) of *Tetranychus urticae* feeding on leaf disks of strawberry cultivars (*Frangaria x ananassa*) (25 ± 1°C; 70 ± 10% RH; 14 h photophase).

Instar	*n*	Strawberry cultivar/ mite development (h)								Bean plant
		Camino Real	Aromas	Camarosa	Diamante	Seascape	Festival	Diamante10	Diamante50	
ED^a^	19	108.9 ± 2.1a*	104.3 ± 3.1ab	102.6 ± 3.0ab	105.1 ± 2.6ab	101.8 ± 3.0ab	102.2 ± 2.6ab	100.5 ± 2.8b	100.1 ± 2.7b	79.4 ± 2.7c
LD^b^	19	24.4 ± 3.3b	29.0 ± 3.4b	46.3 ± 4.3a	29.5 ± 3.5b	31.6 ± 4.0b	25.7 ± 3.8b	23.6 ± 2.8b	23.6 ± 2.6b	20.6 ± 2.2b
PC^c^	19	25.3 ± 2.8a	26.5 ± 2.8a	26.1 ± 3.0a	23.2 ± 2.4a	22.7 ± 3.4a	23.2 ± 3.3a	24.4 ± 2.9a	25.3 ± 3.2a	22.3 ± 2.7a
PND^d^	19	16.0 ± 2.3b	23.2 ± 3.1ab	31.2 ± 3.3a	21.3 ± 2.3b	19.4 ± 2.2b	20.22.7b	18.9 ± 3.2b	16.4 ± 2.5b	12.6 ± 2.2c
DC^e^	19	24.0 ± 2.6a	26.9 ± 3.6a	22.7 ± 3.3a	22.3 ± 2.6a	23.2 ± 2.4a	22.7 ± 2.5a	19.4 ± 2.4a	25.4 ± 2.7a	21.1 ± 2.6a
DND^f^	19	18.9 ± 2.0a	22.3 ± 3.1a	26.9 ± 3.5a	21.5 ± 2.4a	18.9 ± 2.2a	21.9 ± 2.7a	21.9 ± 2.3a	23.2 ± 3.0a	17.3 ± 2.2a
TC^g^	19	27.4 ± 2.7a	31.6 ± 3.6a	29.5 ± 3.6a	31.2 ± 3.5a	36.2 ± 4.2a	26.9 ± 3.3a	27.4 ± 2.9a	32.4 ± 3.7a	27.4 ± 3.7a
Immature	19	244.9 ± 4.8b	263.9 ± 6.6ab	286.4 ± 6.1a	252.9 ± 5.8b	253.8 ± 5.9ab	242.8 ± 5.1b	236.1 ± 4.5b	245.8 ± 6.4b	200.7 ± 4.3c
M^h^	62	(*n* = 11)	(*n* = 7)	(*n* = 4)	(*n* = 9)	(*n* = 8)	(*n* = 10)	(*n* = 9)	(*n* = 4)	(*n* = 9)
		504.7 ± 19.0a	256.0 ± 10.5a	358.0 ± 18.5a	483.6 ± 16.2a	279.0 ± 15.4a	482.4 ± 17.6a	320.0 ± 15.3a	286.0 ± 13.7a	112.0 ± 7.8b
F^i^	90	(*n* = 8)	(*n* = 12)	(*n* = 15)	(*n* = 10)	(*n* = 11)	(*n* = 9)	(*n* = 10)	(*n* = 15)	(*n* = 10)
		370.0 ± 10.9a	334.0 ± 11.9a	285.9 ± 9.8a	312.8 ± 10.5a	421.1 ± 15.4a	414.2 ± 12.7a	464.8 ± 10.8a	349.9 ± 11.1a	207.2 ± 8b
AD^j^	152	448.0 ± 16.9a	305.3 ± 11.5a	301.0 ± 12.9a	393.7 ± 14.5a	361.3 ± 15.5a	450.1 ± 15.7a	396.2 ± 13.8a	336.4 ± 11.7a	162.1 ± 8.9b
Total	304	692.9 ± 17.0a	569.1 ± 11.7a	586.4 ± 12.9a	646.6 ± 14.8a	615.0 ± 15.2a	692.9 ± 15.9a	632.3 ± 13.8a	582.2 ± 11.5a	362.8 ± 8.7b

*Means followed by the same letter do not differ by the Tukey test (*p* > 0.05).

^a^Period of egg duration.

^b^Larval stage development.

^c^Protochrysalis.

^d^Stage duration of protonymph.

^e^Deutochrysalis.

^f^Deutonymph.

^g^Teleiochrysalis.

^h^Male.

^i^Female.

^j^Adult.

The PND on ‘Camarosa’ was significantly longer (49.6%) than on the remaining cultivars (*F* = 8.91, *df* = 8, *p* = <0.001), except for ‘Aromas’ (Table 1). Conversely, no significant difference among the cultivars was observed for the deutonymph stage of development. The chrysalis developmental stage (PCD, DCD, and TCD) was also similar among cultivars, suggesting that during these phases, the substratum did not influence or interfere with the development.

The period for immature development (ID) of *T. urticae* was longer on ‘Camarosa’ (*F* = 9.76, *df* = 8, *p* = <0.001), and the average cycle of the two-spotted spider mite on the tested strawberry cultivars was 53 h longer than that obtained on control plants. AD of *T. urticae* was not different among cultivars, with the AD in ‘Camarosa’ being approximately 19.5% shorter than the average AD (Tables 1 and 2). The AD was inversely proportional to the ID in ‘Camarosa,’ ‘Camino Real,’ ‘Festival,’ and ‘Diamante 10.’ The AD and the male (MD) and female development (FD) on bean leaf disks were shorter than on strawberry leaf disks. The average AD among cultivars was 211.9 h longer than the AD on the control. The average MD was 7.6% higher than the average FD, with those on the strawberry leaf disks, especially on ‘Camarosa,’ struggling with survival. The high variability observed for the FD revealed ‘Seascape’ was not as suitable as the control plant.

**Table 2:** *Tetranychus urticae* mortality (%) and number of individuals that completed the immature phase on strawberry leaf disk (25 ± 1°C; 70 ± 10% RH; 14 h photophase).

Stage	Cultivars of strawberry plant								Plant of reference^b^
	Camino Real	Aromas	Camarosa	Diamante	Seascape	Festival	Diamante 10	Diamante 50	
Egg	8.0 ab^a^	8.0 ab	0.0 b	12. a	8.0 ab	4.0 ab	8.0 ab	0.0 b	14.0
Larva	17.4 bcd	28.3 abc	54.0 a	50.0 a	52.2 a	18.7 bcd	32.6 ab	12.0 cd	7.3
Protochrysalis	5.3 n.s	3.0	0.0	0.0	4.5	0.0	3.2	4.5	7.9
Protonymph	0.0 ns	3.1	8.7	0.0	0.0	7.7	0.0	2.4	8.6
Deutochrysalis	0.0 n.s.	0.0	4.8	0.0	0.0	0.0	3.3	0.0	6.3
Deutonymph	0.0 n.s.	0.0	0.0	0.0	0.0	0.0	3.4	0.0	0.0
Teleiochrysalis	2.8 ab	6.4 a	5.0 ab	0.0 b	4.8 ab	0.0 b	0.0 b	0.0 b	12.5
Adults^c^	35	29	19	22	20	36	28	41	28
Total mortality	30	42	62	56	60	28	44	18	44

*Means followed by the same letter are not statistically different by the Tukey test (*p* > 0.05).

^a^Percentage of spider mites (*n* = 50) that completer the development stage.

^b^Bean plant (*Phaseolus vulgaris*).

^c^Percentage of individuals that reached the adult phase.

The larvae of the two-spotted spider mite had difficulties in getting established on leaf disks of strawberry plants, probably due to the presence of defensive components in the leaf (Rodriguez & Rodriguez 1981). These components can have toxic or anti-digestive effects on the early immature stages of herbivores (Roda & Baldwin 2003), and the high mortality observed for the mite can be related to the plant resistance by antibiosis (Rodriguez & Rodriguez 1981).

The changes observed in the larval and protonymph development of *T. urticae* on ‘Camarosa’ indicate that this variety may induce a higher adaptive cost, and that the observed delay in the development time is also supportive of antibiosis as a mechanism of plant resistance.

The use of detached leaves without the prior presence of herbivores (induced resistance) led to the assumption that constitutive resistance was the only type of resistance that influenced the biology of herbivores, and has been frequently used (Fadini *et al* 2004, Rovenska *et al* 2005).

### *Mortality*

The lowest larval mortality (LM) occurred on ‘Camino Real’ and ‘Diamante 50’ (*F* = 5.37, *df* = 7, *p* = <0.001). The mean LM among the cultivars was 4.5 times greater than in the control. The first instars that survived were adapted to the strawberry disk, and as a consequence, no significant differences were observed for the following instars, except for the TCD, probably due to the high level of adaptability of this polyphagous mite species (Price 1982). The largest number of *T. urticae* that completed their life cycle occurred on ‘Camino Real,’ ‘Festival,’ and ‘Diamante 50’ cultivars.

### *Oviposition*, *longevity*, *and daily oviposition*

Females on ‘Camarosa’ and ‘Diamante’ cultivars had the lowest rate of total oviposition (*F* = 25.54, *df* = 7, *p* = <0.001) (Table 3), but females on ‘Seascape’ were the most fertile, laying 2.2 times more eggs than females on the control.

**Table 3:** Total oviposition, longevity, and daily oviposition (±se) of *Tetranychus urticae* on cultivars of strawberry (*Fragaria x ananassa*).

Treatment	*n*	Total eggs*	Longevity	Eggs/day
Seascape	38	1,796.0 ± 4.34 a	9.9 ± 1.93 ab	5.5 ± 1.55 a
Diamante 10	38	1,327.0 ± 3.96 ab	10.9 ± 1.97 a	3.4 ± 1.22 b
Festival	38	1,008.0 ± 4.17 bc	9.4 ± 1.93 ab	3.2 ± 1.51 bc
Aromas	38	955.0 ± 3.67 bc	11.8 ± 2.13 a	2.4 ± 1.13 bc
Camino Real	38	895.0 ± 3.95 cd	9.8 ± 2.15 ab	2.8 ± 1.36 bc
Diamante 50	38	746.0 ± 3.87 cd	8.0 ± 2.03 ab	2.5 ± 1.36 bc
Diamante	38	545.0 ± 3.28 e	7.4 ± 2.04 b	2.4 ± 1.41 c
Camarosa	38	319.0 ± 2.79 e	8.9 ± 1.86 ab	1.0 ± 0.97 d
Mean	38	948.9	9.6	2.9
Reference^a^	38	826 ± 3.67	4.0 ± 1.38	7.4 ± 2.14

*Means followed by the same letter are not statistically different by the Tukey test (*p* > 0.05).

^a^Bean plant (*Phaseolus vulgaris*).

There was a significant difference in female longevity (*F* = 3.93, *df* = 7, *p* = 0.0004), with those on ‘Diamante’ being the shortest lived ones. The mean longevity on strawberry leaf disks was 2.4 times higher than on control.

**Fig 1:**
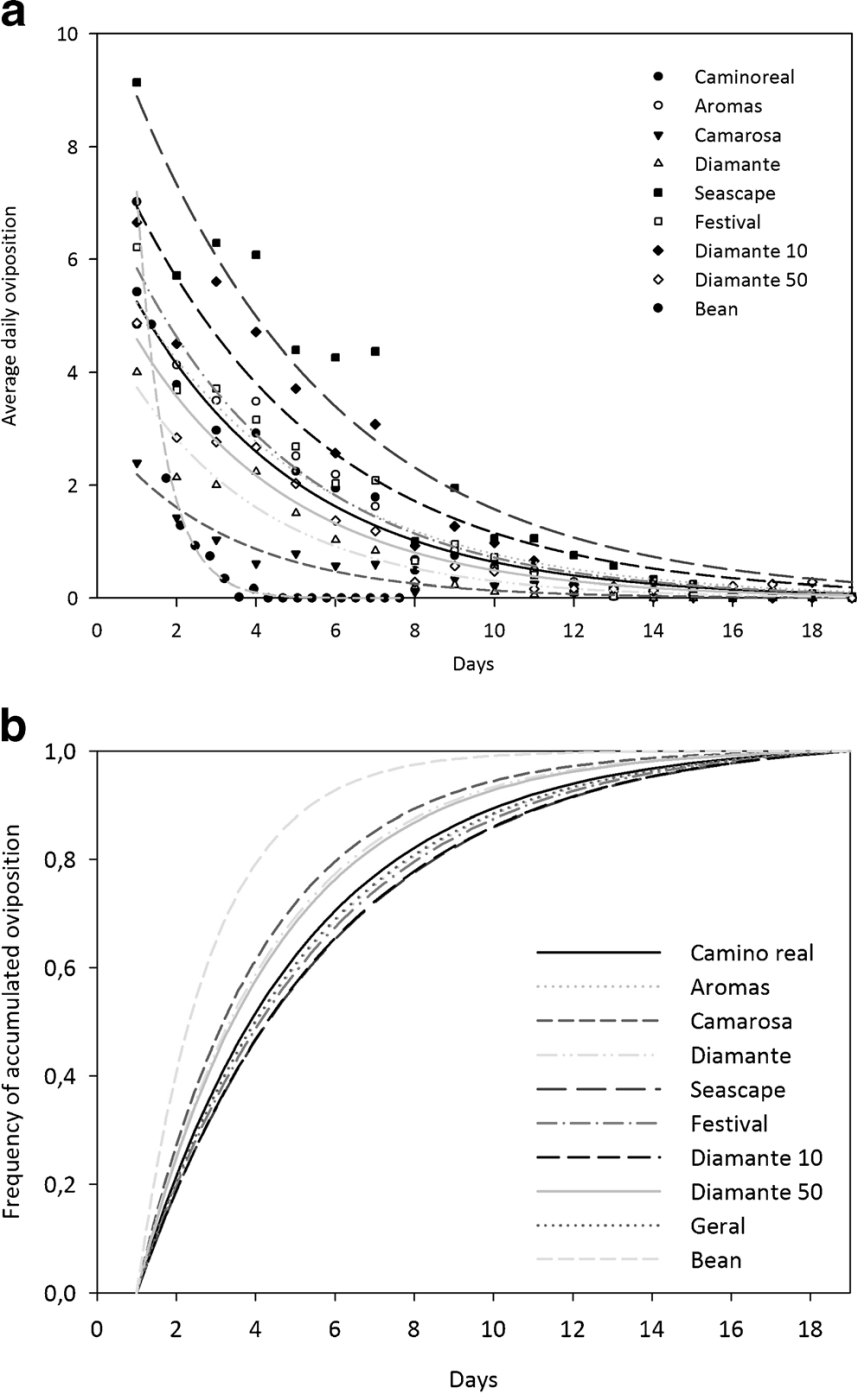
Oviposition of *Tetranychus urticae* on leaf disks of different strawberry cultivars (*Fragaria x ananassa*) and the reference plant (*Phaseolus vulgaris*). **a** Distribution of ovipositions; **b** accumulated oviposition.

The highest daily oviposition was observed for females on ‘Seascape’ (Tables 3 and 4), which was approximately 89.2% higher than the overall average. Eggs were laid following an exponential trend for all cultivars (Fig 1a), and females on ‘Camarosa’ and ‘Diamante’ laid 88.7 and 96.5%, respectively, of the total eggs laid until the eighth day, against the 99.1% on the control (Fig 1b). More than 80% of the eggs of females on the control leaf disks were laid during their mean longevity (3.3 days), while those on ‘Seascape’ and ‘Camarosa’ had laid only 44.8 and 57.7% of their eggs in the same period, respectively (Fig 1b). The distribution of oviposition followed an exponential tendency, presenting a peak of initial oviposition. The curve may indicate an acceleration of reproduction by females due to nutritional deficiency, suggesting that the cultivars tested in this experiment were unfavorable for oviposition. The negative effects of the plant on biology of *T. urticae* were greater in ‘Camarosa’ and ‘Diamante’.

**Table 4:** Temporal exponential model of the mean distribution of *Tetranychus urticae* ovipositions on different cultivars of strawberry (*Fragaria x ananassa*).

Model: Oviposition = a*exp(−b*time)				
Cultivar	a	b	R^2^	*p* value
Camino real	6.24350	0.23742	0.97231	<0.001
Aromas	6.25917	0.22402	0.97536	<0.001
Camarosa	2.28729	0.31530	0.95460	<0.001
Diamante	4.50030	0.29287	0.94513	<0.001
Seascape	11.10774	0.20108	0.93788	<0.001
Festival	6.30423	0.21610	0.95033	<0.001
Diamante 10	7.45394	0.20254	0.92926	<0.001
Diamante 50	6.08298	0.28393	0.96312	<0.001
All	6.19340	0.22618	0.96364	<0.001
Bean plant	12.15035	0.52401	0.98975	<0.001

The effects of cultivars can be associated with the adaptation cost of the herbivores in the adaptation to a new host. This influence was verified by the longer period of immature and adult development of *T. urticae* in all cultivars, as compared with the control. In addition, there was a higher efficiency in the oviposition of females feeding on the bean leaf disks than those feeding on the strawberry leaf disks. Some studies do not consider the adaptation costs of the herbivores in the evaluation of the resistance of plants, especially when these herbivores have been maintained on plants of different species in relation to the ones used in the experiments (Underwood and Rausher 2000, Rovenska *et al* 2005).

We conclude that ‘Camarosa,’ ‘Diamond,’ and ‘Seascape’ were the cultivars that mostly affected the development, survival, and reproduction of *T. urticae* on strawberry crops.
